# Toll-Like Receptor 2 (*TLR-2*) Gene Polymorphisms in Type 2 Diabetes Mellitus

**DOI:** 10.22074/cellj.2019.5540

**Published:** 2018-08-05

**Authors:** Zeynep Ermiş Karaali, Gonca Candan, Mehmet Burak Aktuğlu, Mustafa Velet, Arzu Ergen

**Affiliations:** 1 Department of Internal Medicine, Haseki Training and Research Hospital, University of Health Sciences, Istanbul, Turkey; 2 Department of Molecular Medicine, Aziz Sancar Institute of Experimental Medicine, Istanbul University, Istanbul, Turkey

**Keywords:** Diabetes, Inflammation, Polymorphism, TLRs

## Abstract

**Objective:**

Innate immunity factors are associated with type 2 diabetes (T2DM) and its complications. Therefore, T2DM has been suggested to be an immune-dependent disease. Elevated fasting glucose level and higher concentrations of innate immunity soluble molecules are not only related with insulin resistance, but inflammation is also an important factor in beta cell dysfunction in T2DM. Toll-like receptor 2 (*TLR-2*), which has an important role in inducing innate immune cells, is thought to have suppressive roles on immune responses in T2DM. We therefore aimed to investigate the possible role of *TLR-2* del -196-174 and Arg753Gln variants in T2DM pathogenesis.

**Materials and Methods:**

This study was designed as a case-control study. Polymerase chain reaction-restriction fragment length polymorphism (PCR-RFLP) technique was used to genotype the two variants in 100 T2DM patients and 98 age- matched controls.

**Results:**

We found significantly higher frequencies of *TLR-2* del -196-174 DD genotype (P=0.003), ID genotype (P=0.009) and D allele (P=0.001) in patients compared with controls. In addition, the II genotype (P=0.001) and the I allele (P=0.003) frequencies were elevated in healthy controls. We did not find any significant differences in frequency distribution for the Arg753Gln variant in study groups.

**Conclusion:**

We suggest that carrying the D allele of the *TLR-2* del -196-174 variant may be related as a risk factor for T2DM.

## Introduction

Diabetes is defined by hyperglycemia, resulting from defects in insulin production, activation or secretion. The high prevalence of diabetes mellitus has led to morbidity and mortality (1, 2). The Turkish Diabetes Epidemiology Study II (TURDEP II) has reported the prevalence of diabetes mellitus at 13.7% in 2010 in Turkey (3-5). Previous studies have suggested that innate immune system activation and low-grade chronic inflammation are involved in the pathogenesis of type 2 diabetes mellitus (T2DM) and its complications (6-10).

Toll-like receptors (TLRs) are members of the innate immunity pathway. The role of TLRs on the pathway, which initiate a signal through the nuclear factor kappa B (NF-.B), might have an impact on the development or progression of diabetes (11). In addition, the altered expression and/or function of *TLR-2* may be associated with progression and pathogenesis of immune-related diseases including T2DM and its complications (12-15).

Zhao et al. (16) reported that a high-glucose environment could induce the expression of *TLR-2* and *TLR-4* in retinal ganglion cells, in an attempt to elucidate the role of these genes in the etiology of diabetic retinopathy via increase in the secretion of pro-inflammatory factors. Yin et al. (17) have suggested that the role of inflammation in ß-cell dysfunction may be related through the strong link between TLRs and both inflammation and autophagy. Xiong et al. (18) has shown that the *TLR-2* Arg753Gln variant causes TLR2 signaling-incompetence by effecting to its tyrosine phosphorylation, dimerization and recruitment of Mal and MyD88. *TLR-2* del -196-174 is a 22 bp nucleotide deletion variant that alters the promoter activity of *TLR-2* (19).

The aim of this study was to investigate the possible role of *TLR-2* del -196-174 and Arg753Gln variants in the pathogenesis of T2DM in Turkish patients.

## Materials and Methods

### Case selection

This case-control study was designed at the Haseki Education and Training Hospital, Department of Internal medicine (Istanbul, Turkey). After obtaining written informed consent from the participants and approval from the Ethical Committee of Haseki Education and Training Hospital (No=324/2016), blood specimens were collected in tubes containing EDTA. 

The patient group diagnosed with T2DM consisted of 100 patients comprising 46 females and 54 males (mean age: 73.22 ± 8.30). The control group comprised 98 randomly selected age-matched individuals not diagnosed with T2DM (38 females and 60 males; mean age of 72.84 ± 12.98). 

### DNA isolation

Invitrogen PureLink was used to isolate genomic DNA from the blood samples.

### Genotyping *TLR-2* variants 

*TLR-2* variants were genotyped by using the polymerase chain reaction-restriction fragment length polymorphism (PCR-RFLP) method. A segment of the *TLR-2* gene encompassing the del -196-174 polymorphic site was amplified with specific:

F: 5´-CACGGAGGCAGCGAGAAA-3´
R: 5´-CTGGGGCCGTGCAAAGAAG-3´ (20).
F: 5´-GCCTACTGGGTGGAGAACCT-3´
R: 5´-GCCACTCCAGGTAGGTCTT-3´

specific to a segment containing the Arg753Gln polymorphic site were also synthesised (21). For both variants, PCR reactions were in a total volume of 25 µL containing 200 ng genomic DNA, 10 pmol of each primer, 200 ng dNTPs and 0.6 U Taq DNA polymerase. For the del -196-174 variant, DNA was denatured at 95°C for 2 minutes, followed by 35 cycles of 94°C for 30 seconds, 62°C for 30 seconds and 72°C for 30 seconds, and a final extension step at 72°C for 5 minutes. PCR products were examined by electrophoresis on a 3% agarose gel and the del -196-174 genotypes were identified as I/I= 286 bp, I/D= 286 bp and 264 bp, and D/D= 264 bp. For the Arg753Gln variant, DNA was denatured at 95°C for 2 minutes, followed by 35 cycles of 94°C for 30 seconds, 57°C for 30 seconds and 72°C for 30 seconds, and a final extension step at 72°C for 4 minutes. PCR products were incubated with the AciI restriction enzyme at 37°C for 3 hours and the digested products were visualised by electrophoresis on a 3% agarose gel (GG=228, 75 bp, GA=266, 228, 75 bp and AA=266 bp). 

### Statistical analysis

SPSS 21.0 statistical software package (SPSS, Chicago, IL, USA) was used for statistical analyses. P<0.05 were considered to be statistically significant. We used the χ^2^-test to evaluate the difference in *TLR-2* genotype distribution in the case and control groups. Whenever an expected cell value was less than five, Fisher’s exact test was used. We compared biochemical parameters between the cases and controls by using Student’s t test. Differences in biochemical parameters among the genotypes were investigated by using onne-way ANOVA and Mann-Whitney U test.

## Results

Demographical parameters are shown in Table 1. Distribution of *TLR-2* del -196-174 and Arg753Gln gene variants are shown in Tables 2 and 3. We did not find a significant difference in frequency distribution for the Arg753Gln variant. Nevertheless, del -196-174 DD [P=0.003, 95% confidence interval (Cl)=0.01-0.64] and ID genotypes (P=0.009, 95% Cl=0.06-0.73), and D allele (P=0.001, 95% Cl=0.05-0.43) were found significantly higher frequencies in patients compared with controls. In addition, the II genotype (P=0.001, 95% Cl=1.14-1.46) and I allele (P=0.003, 95% Cl=1.04-1.21) frequencies were elevated in controls. Frequencies of genotypes were not in Hardy-Weinberg equilibrium (del-196-174 in patients, P=0,0001, del-196-174 in controls, P=0.001).

**Table 1:** Demographical parameters in study subjects


Demographical parameter	Patient	Control	P value
	n=100	n=98	
	Mean ± SD	Mean ± SD	

Age (Y)	73.22 ± 8.30	72.84 ± 12.98	>0.05
Gender (F/M, n)	46/54	38/60	>0.05
Fasting blood glucose (mg/dl)	179.35 ± 81.70*	109.81 ± 25.81	<0.001
HbA1c (mg/dl)	7.70 ± 2.67*	4.99 ± 1.91	<0.001
Cholesterol (mg/dl)	172.79 ± 61.13**	130.20 ± 45.21	0.026
Triglyceride (mg/dl)	118.15 ± 93.62	71.67 ± 42.43	>0.05
HDL-cholesterol (mg/dl)	36.66 ± 14.12	34.07 ± 6.69	>0.05
LDL-cholesterol (mg/dl)	107.50 ± 45.98***	74.60 ± 35.69	0.028
C-Reactive protein (mg/L)	63.43 ± 74.72*	19.93 ± 4.42	<0.001
Urea (mg/dl)	80.69 ± 55.69	71.92 ± 66.24	>0.05
Uric acid (mg/dl)	7.18 ± 3.35****	4.54 ± 3.59	0.032
Creatinine (mg/dl)	1.61 ± 1.08*****	1.30 ± 0.86	<0.001
Hypertension (%)	70*	35	0.006


F/M; Female/Male, HbA1c; Hemoglobin A1c, HDL; High density lipoprotein, LDL; Low density lipoprotein, *; P<0.001, **; P=0.026, ***; P=0.028, ****; P=0.006, and *****; P=0.032.

Although we found a significant relationship based on genotype frequencies, there were no relationship between *TLR-2* del -196-174 genotypes and clinical parameters except hypertension in patients. Carrying the II genotype [P=0.034, χ2=4.48, odds ratio (OR)=2.86, 95% Cl=1.06-7.73] and the I allele (P=0.023, .2=5.14, OR=3.99, 95% CI=1.13-14.00) might be a risk factor for hypertension in diabetes. In addition, the DD genotype (P=0.023, .2=5.14, OR=1.77, 95% Cl=1.11-9.23) and the D allele (P=0.034, χ2=4.48, OR=1.45, 95% Cl=1.06-4.16) might have a protective role against hypertension (Table 4). In addition, we observed that HbA1c values were lower in patients with hypertension (7.07 ± 2.24 mg/dl) than non-hypertension (9.10 ± 3.03 mg/dl) patients (P=0.002, 95% Cl=1.74-3.32). 

**Table2:** Distribution of *TLR-2* del -196-174 variant genotypes


*TLR-2* del -196-174 polymorphism	Patient	Control	P value
	n=100	n=98	
	n (%)	n (%)	

II	74 (74%)	94 (95.9%)^*^	0.001
DD	12 (12%)^**^	1 (1%)	0.003
ID	14 (14%)^***^	3 (3.1%)	0.009
I	162 (81%)	191 (95.5%)^**^	0.003
D	38 (19%)^*^	5 (4.5%)	0.001


DD; deletion/deletion genotype, ID; Insertion/deletion genotype, D; Deletion allele, I; Insertion allele, II; Insertion/insertion genotype, *; P=0.001, **; P=0.003, and ***; P=0.009.

**Table 3:** Distribution of *TLR-2* Arg753Gln variant genotypes


*TLR-2* Arg753Gln polymorphism	Patient	Control	P value
	n=100	n=98	
	n (%)	n (%)	

GG	99 (99%)	98 (100%)	>0.05
AA	-	-	-
GA	1 (1%)	-	-
G	199 (99.5%)	196 (100%)	>0.05
A	1 (0.5%)	-	-


**Table 4:** Comparison of del -196-174 genotypes and demographic parameters in T2DM patients


Demographic parameters	del -196-174 genotypes			del -196-174 alleles	
	DD	II	ID	D	I

Fasting blood glucose (mg/dl)	197.50 ± 79.66	175.09 ± 86.43	186.29 ± 55.19	191.46 ± 66.40	176.88 ± 82.10
HbA1c (mg/dl)	7.29 ± 3.19	7.72 ± 2.37	8.12 ± 3.89	7.64 ± 3.42	7.78 ± 2.58
Cholesterol (mg/dl)	182.00 ± 103.26	167.28 ± 58.88	191.17 ± 56.16	188.11 ± 68.25	171.90 ± 58.23
Triglyceride (mg/dl)	105.40 ± 59.55	117.10 ± 91.14	134.00 ± 85.37	121.00 ± 104.69	119.92 ± 89.24
HDL-cholesterol (mg/dl)	40.65 ± 16.65	33.96 ± 12.64	45.93 ± 17.00	44.17 ± 16.03	36.27 ± 14.11
LDL-cholesterol (mg/dl)	106.33 ± 73.07	105.08 ± 43.99	118.17 ± 48.64	114.22 ± 53.37	107.61 ± 44.38
C-Reactive protein (mg/L)	76.58 ± 29.55	64.84 ± 8.79	45.93 ± 15.43	59.41 ± 15.55	61.73 ± 7.78
Urea (mg/dl)	76.30 ± 57.62	78.51 ± 55.61	98.61 ± 55.95	86.97 ± 56.68	81.38 ± 55.74
Uric acid (mg/dl)	6.74 ± 1.95	7.09 ± 3.52	8.19 ± 3.86	7.42 ± 2.97	7.26 ± 3.55
Creatinine (mg/dl)	1.43 ± 1.04	1.55 ± 1.05	2.03 ± 1.21	1.77 ± 1.16	1.63 ± 1.09
Hypertension (%)	41.7^**^	75.8^*^	63.6	52.2^*^	74^**^


DD; Deletion/deletion genotype, ID; Insertion/deletion genotype, D; Deletion allele, I; Insertion allele, II; Insertion/insertion genotype, HbA1c; Hemoglobin A1c, HDL; High density lipoprotein, LDL; Low density lipoprotein, *; P=0.034, and **; P=0.023.

## Discussion

Toll-like receptors are surface proteins on eukaryotic cells to detect and respond to microbial antigens (22). There is evidence for TLRs, especially *TLR-2* and *TLR-4*, that they alter proinflammatory and host defense functions of human neutrophils (23). Expression of *TLR-4* and its ligand confer systemic insulin resistance in condition of elevated free fatty acids in blood (24). 

*TLR-2* is reported to have an important role in the pathogenesis of T2DM in recent studies. For instance, Dasu et al. have shown that *TLR-2* expression is increased in patients with T2DM and contributes to the proinflammatory state (25). Devaraj et al. (26) have reported that *TLR-2* and *TLR-4* ligand levels are increased in type 1 diabetes, resulting in the proinflammatory state of the disease in concert with hyperglycaemia, and thus accounting for the increase in TLR2 and *TLR-4* activity.

Huang et al. (27) reported that SNP rs1898830 in TLR2 is associated with T2DM risk in the Chinese population. They found that the AA genotype was associated with the secretion of pro-inflammatory cytokines and chemokines, which in turn promote inflammation in some tissues, thus resulting in insulin resistance and ultimately T2DM development (28-30). 

Maldonado-Bernal et al. (31) observed that the *TLR-2* R753Q variant was very low in frequency and insignificantly less frequent in T2DM patients than in healthy donors. Wifi et al. (32) found no statistical difference in the distribution of *TLR-2* -1350T/C genotypes in T2DM. In the present study, we have found a significant association for *TLR-2* del -196174 but not for the Arg753Gln gene variant in Turkish T2DM patients. We also observed an association with hypertension, however, to clarify the effect of del -196-174 genotypes on hypertension, study of patients with only hypertension will be essential. 

## Conclusion

Our results demonstrate that the *TLR-2* is associated with increased T2DM risk. Further investigations with larger scale sample sizes are needed to confirm our findings and functional studies will be required to unravel the mechanism underlying this association. 
